# Oxidative Stress: A Key Modulator in Neurodegenerative Diseases

**DOI:** 10.3390/molecules24081583

**Published:** 2019-04-22

**Authors:** Anju Singh, Ritushree Kukreti, Luciano Saso, Shrikant Kukreti

**Affiliations:** 1 Nucleic Acids Research Lab, Department of Chemistry, University of Delhi (North Campus), Delhi 110007, India; anju11278@gmail.com; 2 Department of Chemistry, Ramjas College, University of Delhi, Delhi 110007, India; 3 Academy of Scientific and Innovative Research (AcSIR), CSIR-Institute of Genomics and Integrative Biology (CSIR-IGIB) Campus, Delhi 110007, India; ritus@igib.res.in; 4 Genomics and Molecular Medicine Unit, Institute of Genomics and Integrative Biology (IGIB), Council of Scientific and Industrial Research (CSIR), Mall Road, Delhi 110007, India; 5 Department of Physiology and Pharmacology “Vittorio Erspamer”, Sapienza University of Rome, P. le Aldo Moro 5, 00185 Rome, Italy; luciano.saso@uniroma1.it

**Keywords:** oxidative stress (OS), neurodegenerative disease, reactive oxygen species (ROS), mitochondria, Parkinson’s disease (PD), Alzheimer’s disease (AD)

## Abstract

Oxidative stress is proposed as a regulatory element in ageing and various neurological disorders. The excess of oxidants causes a reduction of antioxidants, which in turn produce an oxidation–reduction imbalance in organisms. Paucity of the antioxidant system generates oxidative-stress, characterized by elevated levels of reactive species (oxygen, hydroxyl free radical, and so on). Mitochondria play a key role in ATP supply to cells via oxidative phosphorylation, as well as synthesis of essential biological molecules. Various redox reactions catalyzed by enzymes take place in the oxidative phosphorylation process. An inefficient oxidative phosphorylation may generate reactive oxygen species (ROS), leading to mitochondrial dysfunction. Mitochondrial redox metabolism, phospholipid metabolism, and proteolytic pathways are found to be the major and potential source of free radicals. A lower concentration of ROS is essential for normal cellular signaling, whereas the higher concentration and long-time exposure of ROS cause damage to cellular macromolecules such as DNA, lipids and proteins, ultimately resulting in necrosis and apoptotic cell death. Normal and proper functioning of the central nervous system (CNS) is entirely dependent on the chemical integrity of brain. It is well established that the brain consumes a large amount of oxygen and is highly rich in lipid content, becoming prone to oxidative stress. A high consumption of oxygen leads to excessive production of ROS. Apart from this, the neuronal membranes are found to be rich in polyunsaturated fatty acids, which are highly susceptible to ROS. Various neurodegenerative diseases such as Parkinson’s disease (PD), Alzheimer’s disease (AD), Huntington’s disease (HD), and amyotrophic lateral sclerosis (ALS), among others, can be the result of biochemical alteration (due to oxidative stress) in bimolecular components. There is a need to understand the processes and role of oxidative stress in neurodegenerative diseases. This review is an effort towards improving our understanding of the pivotal role played by OS in neurodegenerative disorders.

## 1. Introduction

Oxidative stress (OS) is a condition produced by the imbalance between oxidants and antioxidants in a biological system. The imbalance occurs as a result of the excess level of reactive oxygen species (ROS) or improper functioning of the antioxidant system [1]. The crucial role of molecular oxygen in biology is undebatable as it is essential for proper cellular functioning and the life of all the organisms. Although oxygen is crucial for life and is involved in signal transduction, gene transcription, and other cellular activities, coincidently, it also has a deleterious effect on biomolecules in the form of free radical and reactive oxygen species (ROS). The adverse effect of oxygen is the result of its univalent metabolic reduction status, which is responsible for the generation of ROS. The other most important species is nitric oxide (NO), which carries out the regulatory function of relaxation and proliferation of vascular smooth muscle cells, leukocytes adhesion, angiogenesis, platelets aggregation, thrombosis, vascular tone, and haemodynamics, among others [2]. Nitric monoxide (NO•) in its free form is harmful for biomolecules. There are various chemical species comprising of free radicals, containing oxygen such as the hydroxyl radical (HO^•^), superoxide radical anion (^•^O_2_‾), hydroperoxyl radical (HO_2_^•^), and peroxyl radicals (ROO^•^). In the present scenario, “ROS” stands for both oxygen radicals as well as non-radicals that are conveniently converted into radicals (H_2_O_2_, _1_O^2^, etc.) [3,4,5,6]. Halliwell had established that a free radical is a species having one or more unpaired electrons and existing independently on its own [7,8]. Free radicals are supposed to play a substantial role in various biochemical reactions, as well as in biological evolution.

Oxygen is the most important and vital entity for all living organisms including neuronal cells involved in tissue formation, whereas when present in excess, it has deleterious effects. Therefore, it is required that the usage and uptake of oxygen and other essential elements be kept under high security and checked via the complex system of the cell. In mitochondrion, oxygen playsa substantial role in oxidative phosphorylation, involving the breaking down of glucose and generation of cellular energy currency ATP in mitochondria. The enzyme and protein required for the oxidative phosphorylation are encoded by the mitochondrial DNA [9,10]. Any mutation in the mitochondria indirectly leads to the obstruction of oxidative phosphorylation (enzyme and protein synthesis is hampered). Oxygen and other free radicals, produced endogenously (NADPH oxidase, mitochondria, etc.) as well as exogenously (chemicals, radiation, etc.), are constantly added in the cellular environment [11]. The body defense system in the form of antioxidants also co-evolved to combat/counteract the effect of reactive oxygen species. Various antioxidants such as glutathione, taurine, creatine, zinc, vitamin E, vitamin C, vitamin A, and polyphenols from (tea extract) are produced against the ROS. The effect of the antioxidant is enhanced and supported by antioxidant enzymes such as superoxide dismutase, catalase, glutathione peroxidase, and so on [11,12,13]. 

**Figure 1:**
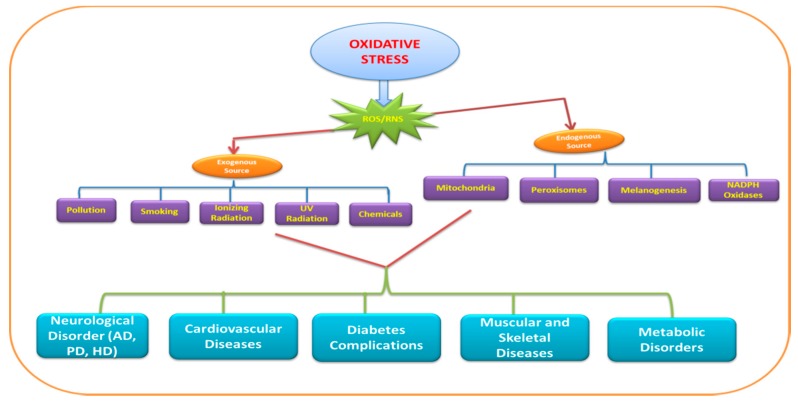
Representation of exogenous and endogenous sources of reactive oxygen species (ROS)/reactive nitrogen species (RNS) anddiseases. PD—Parkinson’s disease; AD—Alzheimer’s disease; HD—Huntington’s disease.

ROS significantly contribute to the deterioration of neuronal cells via modulating the function of biomolecules. These species target different biomolecules (DNA, RNA, lipids, and proteins) and processes (nucleic acid oxidation, lipid peroxidation) in the cell. The brain consumes a large amount of oxygen for proper functioning, andcan be considered as the factory of free radicals/reactive oxygen species (ROS) as well as the hotspot of neurodegeneration. The ROS involved in neurodegeneration include hydrogen peroxide (H_2_O_2_), superoxide anion (O_2_‾), and highly reactive hydroxyl radical (HO^•^). The reactive nitrogen species (RNS) such as nitric oxide (NO) are also found to have a deleterious effect on neurons. Figure 1 depicts the exogenous as well as endogenous sources and diseases caused by ROS/RNS.

The literature is rich in evidence that there is a significant connection between OS and neurodegenerative diseases (Alzheimer’s disease (AD), Parkinson’s disease (PD), Huntington’s disease (HD), Machado–Joseph disease, etc.) as well as ageing [14,15,16,17]. A number of neuroprotective therapies have been developed thus far to combat the ROS protecting neuron cells and obstruct the neurodegenerative diseases. The utmost importance is given to the exact mechanism involved in neurodegeneration and cell death via ROS, as it enables us to find and define the main target for treatment. This review is an effort to compile the mechanisms and causes of neurodegenerative disease and advances made in the study of the substantial role of OS in neurodegenerative diseases and therapies to combat these diseases.

## 2. Sensor and Biomarkers of Oxidative Stress

**Figure 2:**
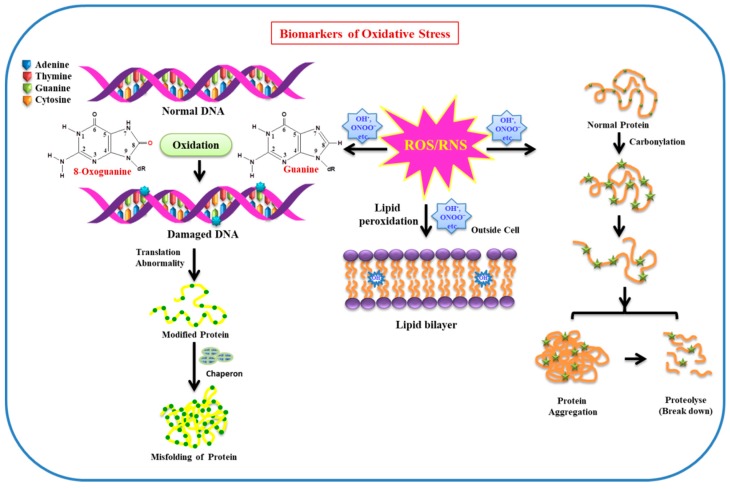
Effect of ROS/RNS on biomolecules (DNA, protein, lipid, etc.) used as biomarkers of oxidative stress in cellular environment.

Owing to the maximum oxygen consumption by the brain (neuron cells), and insufficient antioxidant defense system along with a high content of polyunsaturated lipids, prone to oxidation in neuron cells, OS has a deleterious effect on biomolecules. Biomolecules (lipids, proteins, DNA/RNA, enzymes, etc.) are very sensitive to free radicals (ROS/RNS), so changes and modification to these biomolecules occurring under stress conditions can be used as markers for OS. Lipids and proteins can be functionally altered by various ROS such as OH^•^ and ONOO‾. The heterocyclic bases of DNA/RNA are prone to oxidative damage, in particular, guanine is more susceptible to the attack of ROS, resulting in the formation of 8-hydroxyguanine and 8-hydroxy-2-deoxyguanosine. The increased levels of these modified bases are observed in PD brains, revealing the involvement of OH radicals as oxidative species. Moreover, protein carbonylation and nitration are predominantly observed in AD brains [18,19]. As lipids play a pivotal role in neuronal function in the brain, along with this, they are also found to a larger extent in the plasma membrane, where they act as a barrier between intracellular and extracellular spaces [20]. They are more prone to the attack of free radicals and undergo lipid peroxidation. Owing to lipid peroxidation, membrane fluidity decreases, leading to an increase in membrane leakiness. This facilitates the entrance of those substances to pass in the intracellular system, which usually are unable to cross the barrier except through specific channels (e.g., K^+^, Ca^2+^, etc.). As a consequence, these substances mutilate membrane proteins, enzymes, and receptors, among others [21]. As neuronal membrane lipids are very rich in polyunsaturated fatty acids (PUFA), the side chains are specifically more vulnerable to ROS/RNS, resulting in oxidative stress. Deterioration of these biomolecules can play a substantial role as biomarkers for OS. The effect of ROS/RNS on biomolecules (DNA, protein, lipid, etc.) used as biomarkers of oxidative stress in the cellular environment is illustrated in Figure 2. 

## 3. Mitochondria and Oxidative Stress

**Figure 3:**
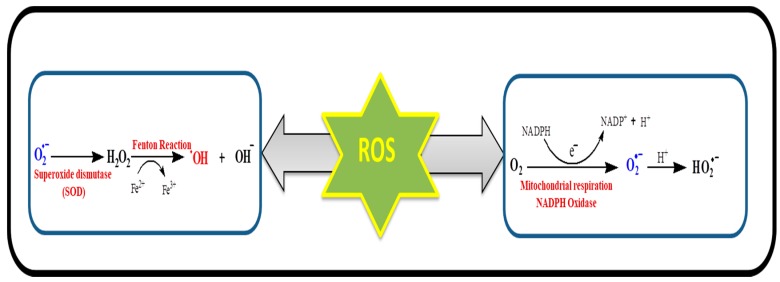
Various reactions for ROS generation. The superoxide (O_2_^•^−) is produced from molecular oxygen (O_2_) in the mitochondria or NADPH oxidase as by-product of respiratory chain. Superoxide can be transformed into hydrogen peroxide via superoxide dismutase. Hydrogen peroxide further leads to the formation of hydroxyl radical and hydroxyl anions.

ATP is essential for the functioning, signaling, and overall activities of the cell and is recognized as the energy currency of the cell. Mitochondria are responsible for the production of ATP through the electron transport chain and oxidative phosphorylation. They are also involved in producing molecules to overcome the oxidative stress along with programmed cell death and other respiratory functions in the cell. Enrichment of mitochondria with various redox enzymes and mitochondrial dysfunction is supposed to be responsible for production of ROS in the cellular environment (Figure 3) [22,23]. 

As evident from literature, the oxidative damage biomarkers of ageing and other neurodegenerative diseases are the biomolecules like lipids, proteins, and DNA. ROS is found to have a deleterious effect on lipids by being involved in lipid peroxidation to malondialdehyde (MDA), protein carbonyls, and oxidation of guanine into 8-oxo-deoxyguanosine in DNA [24,25]. Cardiolipin (CL) is a phospholipid found in the inner mitochondrial membrane and is involved specifically with the proteins of the electron transport chain. It is exclusively required by adenine nucleotide translocase, which functions as an inner membrane transporter. The enrichment of cardiolipin in polyunsaturated fatty acids such as linoleic acid and the location adjacent to the sites of ROS production in the mitochondrial electron transport chain make it a primary target for ROS. CL is prone to oxidation, leads to mitochondrial electron transport chain dysfunction, and is supposed to be involved in the release of proapoptotic proteins [26,27]. 

ROS has a direct damaging effect on proteins and lipids, which hinder the bioenergetics function in mitochondria. It also causes a deleterious effect on mitochondrial DNA, directly associated with promoter inactivation and downregulation of mitochondrial gene expression.

**Figure 4:**
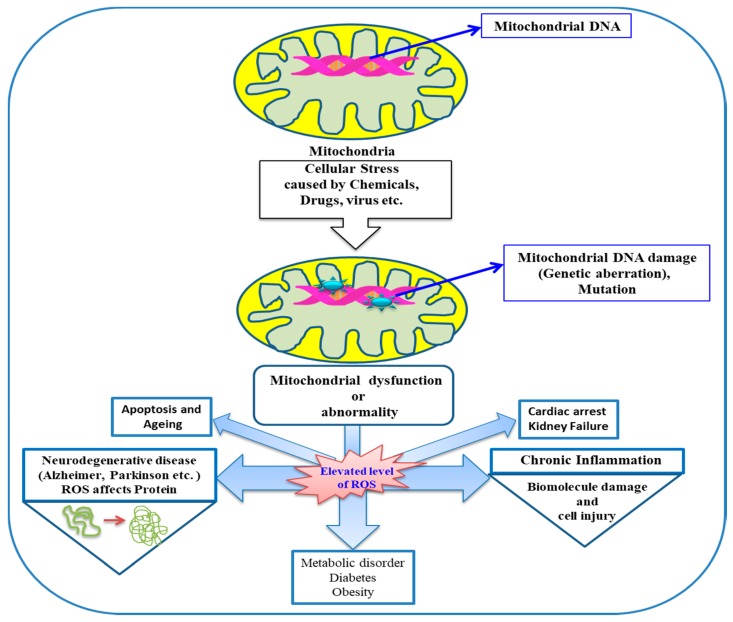
Involvement of mitochondria in oxidative stress and diseases.

Therefore, it is assumed that elevated levels of ROS production in mitochondria, which have longer half-lives—such as H_2_O_2_; lipid hydroperoxide; or active aldehydes like MDA, acrolein, and so on (generated at other locations but reach finally to mitochondria)—can cause mitochondrial dysfunction and ultimately hinder biological processes leading to various diseases. The involvement of mitochondria and mitochondrial dysfunction owing to ROS in various diseases is schematically depicted in (Figure 4).

The brain is highly enriched in oxidizable substrates (PUFA, catecholemics, etc.). The high oxygen demand, relatively less anti-oxidant enzymes, and abundance of catalytic transition metal in some regions of the brain make it more vulnerable to oxidative damage [4,28]. Enormous reports have established that the accumulation of various oxidative damage markers of proteins, lipids, and DNA leads to brain aging [29]. An increased level of 8-OH-dG is observed many folds more in mt DNA than in nuclear DNA. Oxidatively damaged mt DNA leads to various point mutations, which were demonstrated by cloning and sequencing methods [30,31]. A common deletion evidenced in aged persons, found to be accumulated in mt DNA, is the result of oxidative lesions caused by ROS. The main reason for the highest accumulation of this mutation can be the active involvement of the brain in the oxidative catabolism of dopamine. Various DNA glycosylase present in the cell to repair mt DNA are found to decrease in number with ageing, thus leading to somatic mutations in mt DNA in the aged brain [32,33,34]. It is significant to note that oxidative damage caused to mitochondrial proteins and lipids obstructs the bioenergetics functions, but along with this, alterations caused to the mt DNA and nuclear DNA can also affect said functions via the downregulation of protein expression involved in oxidative phosphorylation. 

## 4. Role of Pro-Oxidants in Oxidative Stress

Apart from other exogenous and endogenous sources, various pro-oxidants are also found to play a substantial role in oxidative stress. Reports suggest that fruits and vegetables are a rich source of polyphenols and thus play an important role as efficient antioxidants. This awareness increases the inclusion of these ingredients in our daily routine meals. However, the chronic health problems and neurodegenerative diseases still occurring raise the possibility that besides antioxidants, there may be other factors associated with oxidative stress, later identified as pro-oxidants [35]. Thus, pro-oxidants take in any endobiotic or xenobiotic species, which triggers oxidative stress via producing ROS/RNS or inhibits the functioning of the antioxidant system in cells or tissues. Pro-oxidants may be exogenous and endogenous, which can further be classified in sub-categories—that is, exogenous is divided into pathogens, drugs, toxicants, dietary ingredients, and so on, and endogenous into anxiety, ion flux, climate, environmental pollution, drug metabolites, and so on. 

Some antioxidant flavonoids are reported to behave as pro-oxidants in the presence of some transition metals. It is the flavonoid structure that majorly contributes to the antioxidant properties as well as copper-initiated pro-oxidant behavior. In flavonoids, -OH substitution leads to the antioxidant property of flavonoids, while flavone and flavanone devoid of -OH substitution, facilitating the backbone skeleton of flavonoids, lack antioxidant properties as well as copper initiated pro-oxidant properties [35]. Using variation in the deoxyribose degradation assay, the pro-oxidant and antioxidant properties of flavonoid myricetin were also explored by Chobot et al., [36]. Flavonoids and polyphenols are recognized as having dietary scavenging property for pro-oxidants and facilitate protection against oxidative induced diseases via neutralization of harmful free radical reactions and specifically quenching reactive metals that produce ROS/RNS [37,38]. Likewise, ascorbic acid is also prone to behave as an antioxidant as well as pro-oxidant, in a dose dependent manner. Ascorbic acid may also have a toxic effect owing to its auto-oxidation, leading to influence gene expression [39]. Because it is clear that, along with antioxidants, pro-oxidants also play crucial role in oxidative stress and diseases, the awareness around pro-oxidants is equally important to combat OS and neurodegeneration [40,41].

## 5. Role of Heavy Metals in Oxidative Stress

The role of heavy metals in OS and their harmful effects on the respiratory system; cardiovascular system; reproductive system; central nervous system (CNS); as well as vital organs such as the kidneys, lungs, liver, brain, and so on is undebatable. Various activities cause bioaccumulation of metal ions in the environment as well as in humans [42]. Heavy metals in combination with other xenobiotics like pesticides exert a deteriorating effect on hematology and the immune system. It has been extensively studied that exposure of elevated levels of heavy metals such as lead (Pb) and mercury (Hg), and deficiency of the essential metals like selenium (Se) and Zinc (Zn), lead to oxidative stress. This leads to the abnormality in redox status of the cell, resulting in a deleterious effect on biomolecules (DNA, RNA, proteins, and lipids), as well as vital organs such as the liver, kidney, and CNS [43]. Reports indicate that Mercury (Hg) is not required for any biological process, but its presence and accumulation cause harmful effects in living organisms. Oxidative stress induced by mercury causes membrane damage and oxidation of biomolecules, and also promotes synthesis of H_2_O_2_, lipid peroxidation in the mitochondrial membrane, and protein oxidation [44]. Mercury is also found to be neurotoxic and prolonged exposure to mercury causes a deleterious effect on the brain, resulting in timidity, tremors, memory problems, and changes in hearing and vision [45].

Lead (Pb), a metal used abundantly worldwide, is found to be toxic to humans specifically because of induction of oxidative stress. The damage due to lead depends on the route of exposure, health status, its dose, amount of exposure, as well as age of the subject (organism) [46]. Lead has a high affinity for the –SH group (present in amino acids) and metal cofactors, which results in a reduction of the activity of antioxidant enzymes involved in various biological processes. It is also associated with elevation in oxidative stress and mitochondrial dysfunction, leading to the failure of vital organs [47]. In addition, arsenic (As) is a metalloid also found to be toxic for living organisms, as the prolonged exposure to arsenic leads to carcinogenesis, cytotoxicity, and genotoxicity in humans. Arsenic is also prone to bind with the –SH group of glutathione, which leads to the modification of GSH to GSSG, owing to which H_2_O_2_ is produced [48,49]. Arsenic is involved in the inhibition of glucose absorption in cells, causing gluconeogenesis and fatty acids oxidation. It also causes a harmful effect on the Kreb cycle and thus causes mitochondrial dysfunction. Heavy metals are directly or indirectly involved in the generation of ROS/RNS and lead to oxidative stress, which ultimately deteriorates the mitochondria and biomolecules, leading to cell proliferation, cell differentiation, and apoptosis [50].

## 6. Concept of Ferroptosis and its Implications for Neurodegeneration

It is a well-accepted fact that oxidative stress is one of the major factors linked with neuronal dysfunction and neurodegenerative diseases such as AD, PD, HD, and amyotrophic lateral sclerosis (ALS). Along with other features, a new term ferroptosis (iron-dependent) has emerged recently; to be involved in oxidative stress induced cell apoptosis and neuronal cell damage. By using the biochemical mechanism, Neitemeier et al. has demonstrated that BID (BH3 interacting domain death agonist) plays an important role in oxidative stress mediated mitochondrial dysfunction in ferroptosis [51]. The process of ferroptosis involves the production of soluble and lipid ROS via enzymatic reactions mediating transition metal iron (Fe) [52]. The oxidative stress induced ferroptosis and mitochondrial dysfunction/dysregulation, resulting in neuronal cell death as well as cell apoptosis, has been recently demonstrated in PC12 cells. This study might be extended to solve and throw light on oxidative stress based strategies for protective measures in ND [53]. Further, various groups have been extensively working on the concept of ferroptosis and its involvement in oxidative stress in ND [54,55].

## 7. Role of Oxidative Stress Signaling in Senescence versus Apoptosis

Ageing and cellular senescence are natural processes that lead to degeneration in various biological processes. Various neurodegenerative diseases are caused with the onset of ageing including AD, PD, HD, and ALS. Cellular apoptosis, also known as programmed cell death, is a natural phenomenon, which occurs as a spontaneously regulated process in a biological system [56]. Along with normal ageing, ROS is also one of the major factors in cellular senescence that leads to the increase in the number of senescent cells in tissues on a large scale. As discussed in previous sections, biomolecules act as biomarkers of oxidative stress—they help in the identification of OS assisted cellular senescence and apoptosis [57]. Elevated level of ROS along with a decrease in antioxidants can cause various age-related pathologies and diseases such as cancer, cardiovascular, and neurodegenerative diseases. Exposure to elevated levels of ROS disturbs cellular homeostasis, leading to harmful effects on biomolecules. Cellular senescence can happen to multiple cell types including epithelial cells, lymphocytes, chondrocytes, neurons, and glial cells [58]. OS and mt ROS trigger telomere shortening and dysfunction, and in turn cause cellular senescence. ROS cause a deleterious effect on neuronal cells and thus can have harmful effects on brain. ROS mediated cell signaling is an effective indicator of cell apoptosis [59]. The cumulative effect of ROS and cellular senescence causes ageing and plays a pivotal role in various neurodegenerative diseases (AD, PD, HD, ALS, etc.) [60]. 

Literature is rich in evidence that reflects the role of ROS in initiation and progression of several pathologies including from cardiovascular diseases to neurodegenerative diseases. Various compounds, specifically antioxidants (vitamins, polyphenols, flavonoids, etc.), counteract the effect of oxidative stress and reduce the risk of dreadful diseases [61]. This class of antioxidants draws attention because of having good efficacy in terms of the prevention and treatment of diseases, without any side effects [62,63]. It is the need of the hour to explore more and more natural as well as synthetic antioxidant compounds that can open new avenues for the treatment as well as prevention of diseases caused by OS. 

## 8. Oxidative Stress: Modulator of Neurodegenerative Diseases

Oxygen is a key component of the respiratory system, and is mandatory for the survival of an organism. Oxidative stress harbors various free radicals and molecules derived from molecular oxygen. Though mitochondria are a hub of oxygen and ROS, there exists a homeostatic control in the cell to check the level of ROS under normal conditions. Neurodegenerative diseases are specifically characterized by apoptosis/necrosis and dysfunction of neuronal cells, leading to a malign effect on the neural system. Being the extensively active part of body, the brain is more vulnerable to oxidative stress. The brain has a higher demand for oxygen and thus consumes 20% more oxygen than other parts of body. The brain is also enriched in redox-active metals (copper and iron) that actively participate in ROS generation. As the brain cell membranes are rich in PUFA, they are more prone to lipid peroxidation [64,65]. Although, when present in the optimum concentration, the antioxidant glutathione (GSH) plays a major role in detoxification of ROS species in brain cells. A decreased level of GSH in brain is linked with the elevated level of ROS leading to neurodegenerative diseases such as AD, PD, Huntington’s disease, and Machado–Joseph disease [66,67,68,69,70,71,72].

## 9. Alzheimer’s Disease (AD)

**Figure 5:**
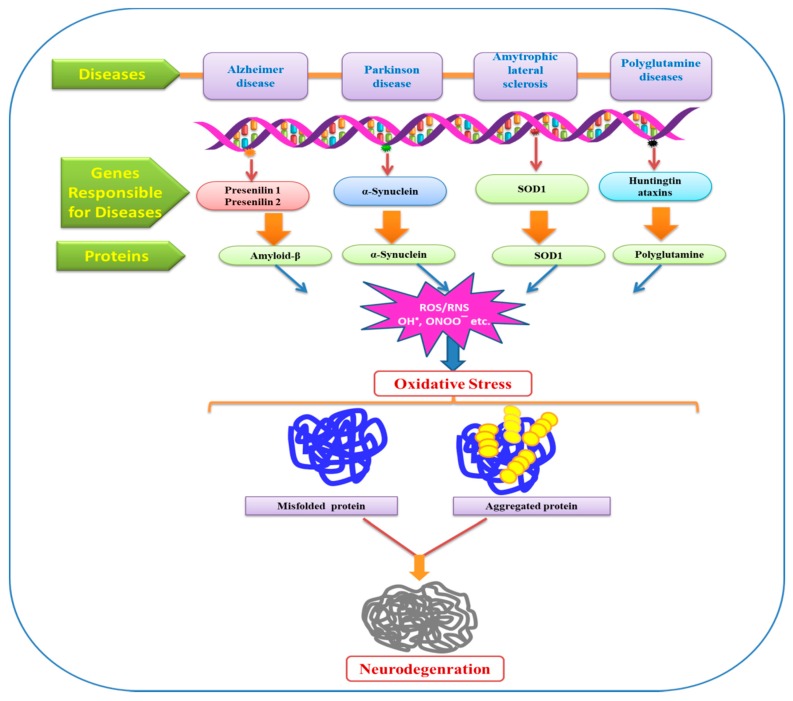
Role of ROS/RNS in oxidative stress thus results in protein damage and various neurodegenerative diseases. SOD—superoxide mutase.

Alzheimer’s disease (AD) or dementia is characterized by progressive loss of cognitive and behavioral deterioration, which leads to the impairment of daily and routine activities. It is one of the most prevalent neurodegenerative disorders manifesting 45 million people worldwide. Usually, AD is called a disease of ageing, but in some cases, it is also observed in the young population. Alzheimer’s disease is manifested with the deposition of protein aggregates, extracellular amyloid plaques (Aβ), intracellular tau (τ) or neurofibrillary tangles, and loss of synaptic connections in specific regions of brain [73,74]. The neuropathological diagnostic feature of AD is the accumulation of neurotoxic Aβ oligomer peptides, which, along with τ protein, mediates neurodegeneration, thus causing neuroinflammation, impairment in synaptic connection, cholinergic denervation, neurotransmitter imbalance, neuronal loss, dendritic alterations, and so on. Massive reports suggest a significant role of ROS and oxidative stress in AD via having a deleterious effect on biomolecules—specifically, proteins (Figure 5). It is evidenced that the oxidative imbalance that leads to the neuronal damage may play a central role in AD. Studies indicate the relationship between Aβ-induced oxidative imbalance and elevated levels of byproducts of lipid peroxidation (e.g., 4-hydroxynonal, malonidialdehyde), protein oxidation (e.g., carbonyl), and DNA/RNA oxidation (e.g., 8-hydroxylguanosine, 8-hydroxyldeoxyguanosine) [75,76,77]. Accumulation of Aβ aggregates is also found to play a pivotal role in OS, which leads to mitochondrial dysfunction and energy failure. Interestingly, decreased levels of antioxidants such as uric acid, vitamin C and E and antioxidant enzymes like superoxide dismutase, catalase, and so on are also observed in AD patients. 

Mounting evidence has suggested that various metals also play a significant role in AD. Metals such as iron, zinc, copper, and so on, which also act as an antioxidant, accumulate in brain as ageing occurs. Various enzymes also require metals for their proper functioning, which is found to be hampered in the AD brain. The role of beta amyloid is still illusive, but it is illustrated to act as an antioxidant, causing hindrance in the formation of hydroxyl radical and thus leading to the prevention of protein and lipid oxidation in mitochondria of rats [78]. Beta amyloid interacts and binds with redox active metals like copper, zinc, and iron, and in this manner, is involved in signaling control of cellular physiology. Beta amyloid undergoes aggregation via complexing with copper in its redox state [79]. Similarly, zinc and iron were found to be involved in microtubule and tau pathology, and it was found that these can bind tau and aggravate aggregation and phosphorylation [80]. Surprisingly, the high concentration of zinc was demonstrated to be interlinked with memory and cognitive regions of the brain—specifically the amygdala, neocortex, and hippocampus are found to be significantly affected in AD patients [81,82].The highly ordered state of Aβ fragment, that is, Aβ (1-40), binds to the zinc and promotes the generation of toxic, fibrillary Aβ aggregates. Subsequently, zinc homeostasis is disrupted in the inflammatory response to non-soluble Aβ plaques, thus resulting in the abnormal release of zinc from cerebrum and enhanced oxidative stress [83]. Apart from the brain, Aβ accumulation is also observed in subcellular areas such as golgi apparatus and endoplasmic reticulum [84]. It is well studied that deposition of beta-amyloid plaque causes major damage, also to mitochondria. The electron transport chain was disrupted along with the production of ROS by beta-amyloid peptide via the inhibition of cytochrome oxidase [85,86,87]. Thus, the connection among mitochondrial dysfunction, tau phosphorylation, and beta-amyloid draws considerable attention towards innovative therapeutic interventions. 

Apart from ROS elevation in AD patients, an increase in RNS is also observed. It is depicted that RNS elevation with modifications is found both in astrocytes as well as in neurons in an AD brain. Gradual modification in astrocyte is identified with an increase in the expression of neuronal (nNOS or NOS-1), cytokine-inducible (iNOS or NOS-2), and endothelial (eNOS or NOS-3) isozymes. The direct association of iNOS and eNOS with Aβ aggregates indicating towards beta amyloid assisted in the induction of nitric oxide synthases (NOS) to produce nitric oxide (NO), which in turn leads to the formation of 3-nitrotyrosine (NT) [88,89]. NO is found to be involved in the promotion of vascular smooth muscle relaxation and thus leads to the proper regulation of blood flow. Elevation in NO is demonstrated as the main cause of generation of ONOO¯, which in turn damages the biomolecules (e.g., lipids, protein, DNA/RNA) [90,91].

It is well documented that oxidative stress and ROS/RNS play a considerable role in the manifestation of AD; however, use of antioxidants to control and prevent AD is still unsettled. The main reason behind this is that most of the antioxidants have permeability limitations, owing to which antioxidants are unable to cross the blood–brain barrier. With the advent of nanotechnology, nanoparticles might prove an efficient vehicle for drug delivery into the CNS [92]. Using multiple antioxidants, their correct dosage, fine-tuned equilibrium in all antioxidants used along with measurements of biomarkers in patients can lead to positive results. Furthermore, in order to understand the exact mechanism for each antioxidant based therapy, more clinical trials are required to design and plan more targeted therapies to combat this cognitive impairment and ultimately AD [93,94]. 

## 10. Parkinson’s Disease (PD)

PD is the second most common neurodegenerative disorder after AD in elderly people characterized by selective neuronal impairment of dopaminergic (DA) neurons in the substantia nigra pas compacta (SNc), along with the reduction of DA levels in the nigrostriatal DA pathway in the brain [95]. The major hallmark of this disease is exhibited by the emergence of insoluble inclusions in neurons known as Lewy bodies, consisting mainly of synuclein. As neurons regulate and control the voluntary movements of the body, their deterioration leads to impaired motor function, bradykinesia, postural instability, rigidity, and tremor at rest. Various exogenous sources such as overuse of herbicides, pesticides, exposure to organic chemicals, carbon monoxide, carbon disulphide, plant derived toxins, and bacterial as well as viral infections are supposed to play a substantial role in the manifestation of PD. It is also believed that ageing plays a pivotal role in PD, as with ageing, normal cellular processes are more prone to ceasing, which leads to degeneration of DAergic neurons [96,97]. The familial forms of PD exhibited various mutations in a number of genes. In order to understand the pathophysiology of PD, it is essential to unveil the mechanism by which mutation led to degeneration of nigralneuron [98].

Several reports indicate that the involvement of ROS and oxidative stress might be one of the major factors causing PD (Figure 5). The exact pathway and mechanism of PD is still incredulous, but it is believed and demonstrated in many reports that in particular, the substantia nigra of PD patients are found to have elevated levels of oxidized lipids, proteins, and DNA, along with reduced levels of glutathione [99,100,101,102]. It is also observed that inflammatory markers such as tumor necrosis factor (TNF)-α; interleukin (IL)-1β, IL-6, IL-10, and so on, and transforming growth factor (TGF)-β in microglia (the main immune cells in brain) were suppressed and non-functional in PD patients [103]. The presence of high levels of trace element (Fe^++^) aggravates cell damage occurring in substantia nigra owing to lipid peroxidation. Along with ferrous ion, other trace elements (e.g., manganese, zinc, selenium, copper, aluminum, etc.) are also found to be involved in neurodegeneration [104,105]. Apart from ROS, it is evidenced that RNS also plays major role in nitrosative stress. NO is generated by NOS, which is found in large quantities in cells, as well as in the extracellular space around dopaminergic neurons produced by nNOS or iNOS [106]. NO obstructs various enzymes in addition to complex I and IV of the mitochondrial electron transport chain, resulting in elevated levels of ROS. Additionally, it hinders the function of proteins by forming S-nitrosothiols, and mediates lipid peroxidation, thus causing a deteriorating effect in PD brains. In situ hybridization and immunohistochemical studies also established the role of NO in PD via postmortem brain tissue analysis, which indicates an elevated level of iNOS and nNOS in basal ganglia structures [107,108]. 

Interestingly, various proteins are also found to play a crucial role in familial forms of PD. PTEN induced putative kinase I (PINK 1), Parkin, DJ-1, Leucine-rich repeat kinase 2 (LRRK2), and α-synuclein are the proteins that displayed their link with PD [109,110,111,112,113]. Among these proteins, α-synuclein is found to have strong association with PD, owing to its presence in Lewy bodies, which develop and aggregate in the course of progression of PD [114]. Proteins PINK 1 and Parkin are associated with autophagy for damaged mitochondria, whereas functional impairment of these proteins leads to accumulation of damaged mitochondria in cells [115,116].DJ-1 is found to play as a marker and sensor for oxidative stress, as well as a redox-chaperone protein [117,118]. It is also evidenced that neurons with mutations in protein LRRK2 are more vulnerable to mitochondrial toxins [119]. 

There are various factors involved in the progression of neurodegeneration leading to PD; however, the exact mechanism is still illusive. Although PD involves a cascade of events thatultimately play a substantial role in the aggravation of the disease, it is important to decipher the exact pathway via which this disease progression can be stopped. Various genetic as well as environmental factors are involved in PD, for example, mutation in various proteins is shown to be involved in mitochondrial perturbation as well as to enhance oxidative stress. Metal mediated toxicity also plays a crucial role in the elevation of ROS, enhancing oxidative stress in cellular environment and causing damage to neurons. The main challenge in PD pathology is the varied complexity of an individual case; therefore, a substantial case study (sample size) will be required for better understanding of PD. Appropriate antioxidants should be identified as therapeutic candidates for the treatment. Like the AD, the main hurdle to be resolved in treatment of PD is the ineffective drug delivery, restricted because of the blood–brain barrier. Mitochondrial DNA deletions are identified in pigmented neurons present in substantia nigra of aged and PD brains. Additionally, the morphology and mitochondrial function concomitantly deteriorate with advancing age, leading to the accumulation of defective mitochondria. Recent advances in *in vitro* studies have opened new avenues to understand PD pathology and have illuminated novel insights to develop therapeutic candidates to target the reasons for PD.

## 11. Amyotrophic Lateral Sclerosis (ALS)

Among the various neurodegenerative diseases, amytrophic lateral sclerosis (ALS) is the most common type of motor neuron disease; it is sometimes called Lou Gehrig’s disease, after the famous baseball player who had this condition. ALS is a fatal neurodegenerative disorder characterized by the progressive degeneration of upper and lower motor neurons in the spinal cord, cortex, and brainstem [120]. It was found that approximately 10% of familial ALS (FALS) cases are the result of mutation in the gene responsible for encoding the superoxide dismutase, whereas 90% of ALS cases are sporadic (SALS), that is, has no clear genetic connection (Figure 5). The most common genetic mutation is identified as expanded GGGGCC hexanucleotide repeat in the non-coding region of the *C9Orf72* gene located on chromosome 9p21 [121]. Various reports indicated the involvement of several factors in ALS, such as excitotoxicity, mitochondrial dysfunction/dysregulation, endoplasmic reticulum stress, neuroinflammation, and oxidative stress [122]. However, the interlinking of these events in this complex disease is still a puzzle. OS mediated protein injury, lipid peroxidation, and DNA and RNA oxidation has been observed in ALS patients. Even OS biomarkers are profoundly found in SALS patient’s urine, cerebro spinal fluid (CSF); blood; and individual tissues or in combination with one or more biomolecules such as elevation in malodialdehyde (MDA) modified protein, nuclear DNA 8-hydroxy-2’-deoxyguanosine (8-OHdG), and lipid peroxidation product [121]. Numerous studies are ongoing in parallel to understand and treat ALS; however, the etiology of the disease still stands unrevealed. As far as ALS treatment is concerned, there has been only a single FDA (Food and Drug Administration) approved drug, namely Riluzole, that has been proven to act as an antagonist to glutamate [123].Various antioxidants were used to scavenge free radicals, research has shown that vitamin E in combination with PUFA reduced the risk of ALS development. Edavarone (MCI-186) is also investigated as a free radical scavenger and it was demonstrated that it efficiently removes lipid peroxide and hydroxyl radicals via transferring electron to the radical, leading to a protective effect on neurons. Edavarone is also shown to eliminate nitrosative stress in the CSF of ALS patients [124].Other antioxidants such as acetylcysteine, creatine, and so on were not found to be effective in the progression of ALS [125].

The involvement of mitochondrial damage along with other factors in ALS is well documented. Mitochondrial dysfunction and OS are directly interlinked with each other, which assists in the elevation of ROS/RNS. ROS can cause mitochondrial DNA mutations, membrane permeability, and calcium homeostasis, as well as enhance the oxidation of lipids and protein carbonylation, thus leading to various neurodegenerative diseases including ALS [126,127]. Research is active worldwide working on ALS to uncover its exact mechanism of pathogenesis and the factors involved [128,129,130]. Ongoing research may provide hope and insights about the exact mechanism and pathways of the disease to develop drugs as well as a therapeutic target for the ALS. 

## 12. Huntington Disease (HD)

Huntington’s disease (HD), named after George Huntington in 1872, is a fatal and autosomal dominant inherited progressive neurodegenerative disorder resulting in neuronal degeneration in the striatum followed by deterioration of the cerebral cortex and thalamus. HD is caused by a mutation in the *huntingtin (HTT)* gene. It is characterized by an abnormal extension in the cytosine–adenine–guanine (CAG) repeat in this gene, which in turn translates into an abnormally long repeat of polyglutathione in the mutant huntingtin protein (Figure 5).Huntington disease is mainly characterized by impaired motor and cognitive traits, personality change, and psychiatric illness [131]. HD usually occurs in midlife falls in between the ages of 35 to 50 years, progresses over period of time, and turns fatal after first symptoms. In HD patients, motor functions are affected initially, while cognitive impairment and profound dementia can be observed at later stages [132]. While it has been demonstrated that the primary cause of HD is toxicity of the mutant *HTT*, various other factors and processes are also found to be associated with the disease. Protein misfolding, abnormal proteolysis, protein aggregation, transcriptional dysfunction, excitotoxic and oxidative stress, and glial activation has also been associated with neuronal death in HD [133]. It was discussed in the previous section how the brain is vulnerable to ROS/RNS and how ROS/RNS affect neurodegeneration. Reports have demonstrated the involvement of oxidative stress and mitochondrial dysfunction mediated neuronal degeneration in HD [134]. Like other NDs, biomolecules play a substantial role as biomarkers for OS in HD patients. Lipid peroxidation, DNA damage, and specifically protein carbonylation were found to be more pronounced in HD [135]. Free radicals have an oxidative effect at intra as well as extracellular levels and trigger DNA oxidation, intracellular protein, and membrane lipid peroxidation. Oxidative DNA damage induces DNA repair pathways, leading to the removal of oxidized bases and restoration of the normal structure as well as function of DNA. It has been shown that repairing damaged DNA might lead to the expansion and instability of CAG trinucleotide repeats in mutant Huntingitin [136].

In view of the involvement of oxidative stress as the key regulator and progression of HD, various therapies may be adapted to combat OS. These include antioxidant therapies (enzymatic as well as non-enzymatic) to scavenge ROS in neurons [137,138].Enzymatic antioxidants include superoxide mutase (SOD), glutathione peroxidase (Gpx), and catalase (CAT), whereas non-enzymatic antioxidants include ascorbic acid (vitamin C), α-tocopherol (vitamin E), glutathione (GSH), retinoic acid, carotenoids, flavonoids, and so on [139]. Despite the fact that HD is an extensively studied neurogenerative disease, and that the role of OS is well detected therein, the exact pathogenesis and pathways of HD are still unclear. More elaborate studies in the future will certainly help in understanding the exact mechanism involved in HD and provide insight into drug development as well as revelation of therapeutic targets [140].

## 13. Conclusions and Outlook

Increased oxidative stress has been viewed as one of the potential common etiologies in various neurodegenerative diseases. Literature is rich with evidence of the involvement of free radicals (ROS/RNS) in the pathophysiology of these health disorders. Normally, a fine balance between the presence of ROS and antioxidants is essential for the proper normal functioning of the cell. ROS generation takes place at varied locations in the cell, especially in mitochondria. Antioxidants combat oxidative stress by working to neutralize free radicals and inhibiting them from initiating the chain reactions that lead to various health disorders and premature aging. Under normal conditions, the presence of a natural antioxidant system actively participates in scavenging ROS and maintains the typical cellular environment. The onset of oxidative stress produces ROS, which have a deleterious effect on the biomolecules causing lipid peroxidation, protein misfolding and aggregation, DNA damage, and mutations. Owing to the high consumption of oxygen and enrichment in PUFA, the brain is the most vulnerable part of the body. ROS cause a damaging effect on neurons and accumulate in the brain, resulting in neurodegenerative diseases. Though metals are crucial for the enzyme mediated reactions in cellular metabolism and cell signaling, mutation in mitochondrial DNA and metal overload in the aged brain subsequently lead to oxidative stress. A cascade of events takes place that ultimately impair neuronal proteins, leading to neuro-inflammation and neurological disorders manifested in loss of cognitive function (AD, PD, ALS, and HD). Mitochondrial dysfunction also plays a substantial role in the imbalance in ROS and the antioxidant system in the cellular environment. 

In the present scenario, research is believed to focus on the development of antioxidants that can efficiently scavenge free radicals and combat oxidative stress. The foremost impediment in ways of delivery of antioxidant/drugs is the blood–brain barrier, posing selective permeability to a specific set of substances only. Several antioxidant therapeutic targets are developed that are capable of neuroprotection prior to OS, preventing free radical production as well as modulating normal metal homeostasis. Additionally, antioxidants are formulated to cure neuronal inflammation as well as free radical scavenging [141]. Interestingly, saffron has been found to act as an antioxidant, playing a key role in the protection of CNS diseases. Low cytotoxicity, commercially availability, and ability to cross the blood–brain barrier make it a suitable candidate to combat various diseases [142]. To stop the degeneration of neurons in the brain, stem-cell oriented therapy is the sole hope for regional reconstruction. Thus, severe nerve damage should be controlled via balancing the ROS generation and its scavenging by antioxidants. Although pre-clinical studies have shown promising results, the benefit of antioxidant therapy for neurodegenerative diseases is still controversial in humans [143].

Following the notion ‘prevention is better than cure’ should help in delaying the neurodegeneration. Ageing and lifestyle are other major factors that play a key role in the onset of neurodegenerative diseases. However, changing one’s lifestyle (continuous physical and cognitive activity), following balanced diet (along with vitamin C and E), developing efficient antioxidants, and early diagnosis may also assist in the treatment of neurodegenerative disorders. Ongoing research worldwide is opening new avenues and hope for future therapeutic targets to control these neurodegenerative diseases. Further studies would also deepen the translational impact of phenomenon of oxidative stress into biology and medicine.
